# Effects of CYP3A Inhibition, CYP3A Induction, and Gastric Acid Reduction on the Pharmacokinetics of Ripretinib, a Switch Control KIT Tyrosine Kinase Inhibitor

**DOI:** 10.1002/cpdd.1110

**Published:** 2022-05-13

**Authors:** Xiaoyan Li, Mark J Shelton, Jing Wang, Julie Meade, Rodrigo Ruiz‐Soto

**Affiliations:** ^1^ Deciphera Pharmaceuticals LLC Waltham Massachusetts USA; ^2^ Certara Princeton New Jersey USA

**Keywords:** drug–drug interaction, ripretinib, CYP3A inducer, CYP3A inhibitor, proton pump inhibitor

## Abstract

Ripretinib is a switch control KIT kinase inhibitor approved for treatment of adults with advanced gastrointestinal stromal tumors who received prior treatment with 3 or more kinase inhibitors, including imatinib. Ripretinib and its active metabolite (DP‐5439) are cleared mainly via cytochrome P450 enzyme 3A4/5 (CYP3A4/5), and ripretinib solubility is pH‐dependent, thus the drug–drug interaction potentials of ripretinib with itraconazole (strong CYP3A inhibitor), rifampin (strong CYP3A inducer), and pantoprazole (proton pump inhibitor) were each evaluated in open‐label, fixed‐sequence study designs. Overall, 20 participants received ripretinib 50 mg alone and with itraconazole 200 mg once daily, 24 participants received ripretinib 100 mg alone and with rifampin 600 mg once daily, and 25 participants received ripretinib 50 mg alone and with pantoprazole 40 mg once daily. Ripretinib exposure increased with concomitant itraconazole, with geometric least‐squares (LS) mean ratios of ripretinib area under the concentration–time curve from 0 to ∞ (AUC_0–∞_) and maximum observed concentration (C_max_) of 199% and 136%. Ripretinib exposure decreased with concomitant rifampin: geometric LS mean ratios for ripretinib AUC_0–∞_ and C_max_ were 39% and 82%. Pantoprazole coadministration had no effect on ripretinib pharmacokinetics. No unexpected safety signals occurred. No dose adjustment is required for ripretinib coadministered with gastric acid reducers and strong CYP3A inhibitors; patients also receiving strong CYP3A inhibitors should be monitored more frequently for adverse reactions. Concomitant ripretinib use with strong CYP3A inducers should be avoided. Prescribers should refer to approved labeling for specific dose recommendations with concomitant use of strong and moderate CYP3A inducers.

Gastrointestinal stromal tumors (GISTs) are a rare heterogenous sarcoma; however, they are the most common sarcoma tumor of the gastrointestinal tract, with a worldwide incidence of approximately 10 to 15 per million.^1^, ^2^ GISTs commonly contain oncogenic mutations in the receptor tyrosine kinase KIT or platelet‐derived growth factor receptor α (PDGFRA).^3^, ^4^, ^5^ Ripretinib is a switch control tyrosine kinase inhibitor that broadly inhibits KIT and PDGFRA signaling by durably binding the switch pocket and activation loop, securing the kinase into the inactive formation.^6^ It is indicated for the treatment of adult patients with advanced GIST who have received prior treatment with 3 or more kinase inhibitors, including imatinib. The recommended dosage approved in the United States, Canada, Australia, China, Hong Kong, Taiwan, and the European Union is 150 mg once daily with or without food.

Ripretinib pharmacokinetics have been characterized in cancer patients.^7^ Ripretinib is systemically absorbed and reaches maximum plasma concentrations approximately 4 hours after a single dose.^7^ For ripretinib, the area under the curve (AUC) is generally proportional to dose from 20 mg once daily up to 150 mg once daily, but C_max_ is less than dose proportional.^7^ Metabolite identification studies in hepatocytes revealed that the major metabolic pathway of ripretinib is N‐demethylation.^6^ The resulting active metabolite, known as DP‐5439, has a similar inhibition profile compared to parent ripretinib.^6^ Steady‐state conditions for ripretinib are reached within 14 days; the accumulation ratio of ripretinib is approximately 1.7 following once‐daily dosing of 150 mg, whereas the accumulation of DP‐5439 was approximately 5‐fold at steady‐state.^7^ Human urine and fecal samples collected from 10 healthy participants following the administration of a single dose of ripretinib 50 mg showed both ripretinib and DP‐5439 are eliminated minimally renally (<1% excreted in urine combined) and were the primary components excreted in feces (data on file). Metabolite profiling of human plasma from these 10 healthy participants also showed ripretinib and DP‐5439 were the primary components identified in plasma (data on file).

In vitro studies with recombinant human cytochrome P450 isoforms (CYPs) and human liver microsomes (with chemical inhibitors) indicate that CYP3A4/5 plays a major role in the metabolism of ripretinib (data on file). When incubated with a panel of recombinant human CYPs, ripretinib (0.1 or 1 μM) disappeared in the presence of CYP3A4 (87.8%–100%), CYP2D6 (97.7%–100%), CYP2C8 (71.2% and 18.9%, respectively), and CYP2C19 (15.7% and 6.7%, respectively). Incubations with the other recombinant human CYP enzymes evaluated (CYP1A2, CYP2B6, and CYP2C9) resulted in less than 7% substrate disappearance. In a further incubation of ripretinib (0.1 μM) with human liver microsomes (0.5 mg protein/mL) for 30 minutes, inhibition of CYP3A4/5 (with ketoconazole) and CYP2D6 (with quinidine) decreased metabolism of ripretinib by up to 63% (CYP3A4/5) and 26% (CYP2D6). When metabolism‐dependent inhibitors were evaluated, inhibition of CYP3A4/5 (with troleandomycin) inhibited metabolism of ripretinib by 79%, whereas inhibition of CYP2C8 (with gemfibrozil glucuronide) inhibited metabolism of ripretinib by 24% and inhibition of CYP2D6 (with paroxetine) inhibited ripretinib metabolism by only 16%. These results strongly indicate CYP3A4 is the major metabolizer of ripretinib, although CYP2C8 and CYP2D6 also play a role in metabolism to a lesser extent. Similar in vitro studies indicated that CYP3A4/5 also plays a major role in the metabolism of DP‐5439 (data on file).

As ripretinib is used to treat patients with advanced cancer, it is expected to be coadministered with many other drugs, including known CYP3A4 inhibitors such as antifungals (eg, itraconazole) and inducers such as rifampin, carbamazepine, and phenytoin. It is therefore important to understand the potential for drug–drug interactions (DDIs) between ripretinib and other drugs that are strong CYP3A inhibitors or inducers. Ripretinib may also be subject to drug interactions with acid‐reducing agents (ARAs) due to its pH‐dependent solubility. Proton pump inhibitors (PPIs) such as pantoprazole not only are one of the most extensively prescribed ARAs in patients with GIST but also cause worst‐case DDI liability between ARA and weakly basic drugs with pH‐dependent solubility. Because PPIs inhibit secretion of gastric acid to a greater extent and with a longer duration than other ARAs, such as H2 receptor antagonists and antiacids, they are more commonly prescribed for long‐term usage for chronic conditions such as gastroesophageal reflux disease or gastric ulcer.^8^ The long‐term blockade of gastric acid secretion decreases the solubility of concomitantly administered weakly basic drugs and interferes with subsequent absorption in intestine.^9^ Overall, changes in the metabolism or absorption of ripretinib could influence its efficacy and safety and are important to identify for clinical treatment.

## Methods

### Ethics Statement

The protocols were reviewed and approved by IntegReview (Austin, Texas). All participants provided written informed consent prior to enrollment, and studies were performed in accordance with ethical principles of Good Clinical Practice and the Declaration of Helsinki. These studies were carried out at Prism Clinical Research, LLC, in Minneapolis, Minnesota. All three studies assessed the potential of drug interactions to influence ripretinib and active metabolite DP‐5439 exposure according to US Food and Drug Administration (FDA) and European Medicines Agency guidance on DDIs.

### Study Design

All studies were phase 1, fixed‐sequence, open‐label studies in healthy volunteers. One study consisted of two separate cohorts examining DDIs between ripretinib and itraconazole or pantoprazole, respectively; the rifampin study was conducted separately. Studies and cohorts were enrolled independently. Eligible participants were healthy male or female adults between 18 and 55 years of age with a body mass index (BMI) of ≥18.5 and ≤30 kg/m^2^. Good health was confirmed by a physician and based on medical evaluation with no significant findings, including medical history, physical examination, laboratory tests, and 12‐lead electrocardiogram (ECG) at screening and day −1. Participants were required to abstain from alcohol, marijuana, grapefruit, Seville oranges, tangelo, star fruit, and grapefruit‐like products 14 days prior to and during the study. Participants with a positive breath test for *Helicobacter pylori* were excluded from the pantoprazole study.

#### Itraconazole (Strong CYP3A Inhibitor)

**Figure 1:**
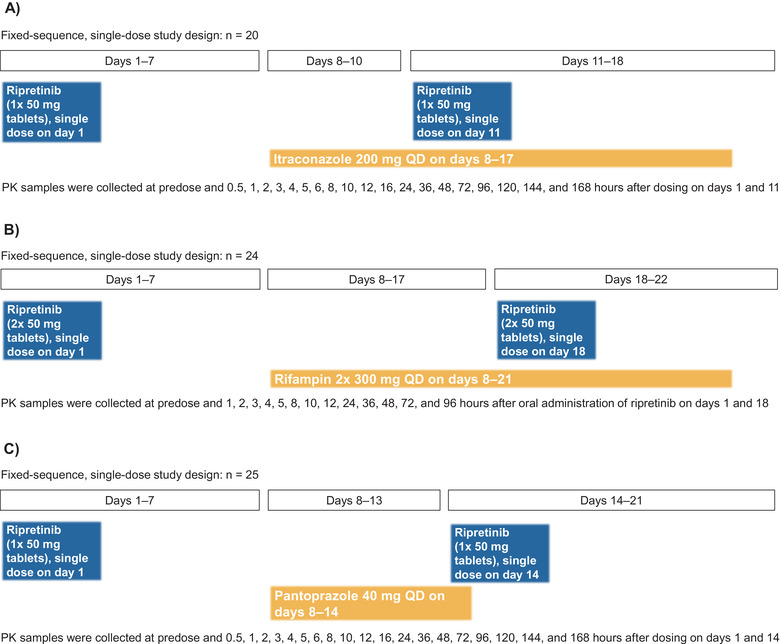
Study design of ripretinib (A) DDI with itraconazole, (B) DDI with pantoprazole, and (C) DDI with rifampin. DDI, drug‐drug interaction; PK, pharmacokinetics; QD, once daily.

Enrollment was staggered to ensure acceptable tolerability, with at least 24 hours elapsing between dosing of the first and second groups of participants. Participants received study drug over 17 days (Figure 1A) and were confined to the study center on days 1–3 and days 8–18. On day 1, participants received a single 50‐mg oral dose of ripretinib under fasting conditions; a lower dose of ripretinib relative to the clinical dose was used due to the potential for increase in ripretinib exposure when coadministered with a strong CYP3A inhibitor. On days 8 through 10, participants received itraconazole 200 mg once daily with a meal or snack. On day 11, participants received ripretinib 50 mg 1 hour after itraconazole 200 mg under fasting conditions. On days 12 through 17, participants received itraconazole 200 mg once daily with a meal or snack. Blood samples for pharmacokinetic (PK) analysis were collected at predose and 0.5, 1, 2, 3, 4, 5, 6, 8, 10, 12, 16, 24, 36, 48, 72, 96, 120, 144, and 168 hours after dosing on days 1 and 11. Safety was assessed throughout and a follow‐up safety phone call was conducted at day 24 or 7 days after the last dose of study drug if participants withdrew early.

#### Rifampin (Strong CYP3A Inducer)

Participants were confined to the study center from days −1 through 22 for the collection of plasma PK samples and all study procedures. Participants received an oral dose of ripretinib 100 mg on day 1 under fasting conditions; a higher dose of ripretinib was used than in the itraconazole or pantoprazole study due to the potential for decreased ripretinib exposure when coadministered with a strong CYP3A inducer (Figure 1B). On day 8, participants received oral doses of rifampin 600 mg once daily 1 hour before a meal with a full glass of water for 14 consecutive days through day 21. On day 18, participants received a second dose of ripretinib 100 mg administered simultaneously with rifampin 600 mg under fasting conditions. Blood samples for PK analysis were collected at predose and 1, 2, 3, 4, 5, 8, 10, 12, 24, 36, 48, 72, and 96 hours after administration of ripretinib on days 1 and 18. Safety was assessed throughout the study period and with a follow‐up phone call on day 29 or 7 days after discontinuation in the case of early withdrawal.

#### Pantoprazole

Participants were confined to the study center on days 1 through 16. On day 1, participants received an oral dose of ripretinib 50 mg under fasting conditions (Figure 1C). On days 8 through 13, participants received pantoprazole 40 mg once daily. On day 14, participants received pantoprazole 40 mg once daily and ripretinib 50 mg simultaneously under fasting conditions. PK samples were collected at predose and 0.5, 1, 2, 3, 4, 5, 6, 8, 10, 12, 16, 24, 36, 48, 72, 96, 120, 144, and 168 hours after dosing on days 1 and 14. Safety was assessed throughout the study period and with a follow‐up phone call on day 28 or 7 days after the last dose of study drug if participants withdrew early.

### Bioanalytic Methods

Ripretinib and its active metabolite DP‐5439 plasma concentrations were measured using a validated high‐performance liquid chromatography (HPLC) with tandem mass spectrometry (LC‐MS/MS) assay with a lower limit of quantification (LLOQ) of 2.00 ng/mL for both analytes. In this method, the analytes (ripretinib and DP‐5439) and the internal standards (ISs; DCC‐2618‐d_5_ and DP‐5439‐d_5_) were released from human plasma samples by protein precipitation. The supernatants were chromatographed using reversed‐phase HPLC with a Betasil® C_8_ analytical column with deionized water (0.1% formic acid) and 40/60 acetonitrile/acetone (0.1% formic acid) as the mobile phase. Mass spectrometric detection was performed on a triple quadrupole tandem mass spectrometer API 5000 or API 5500 (Applied Biosystems/MDS Sciex, Toronto, Canada) equipped with a turbo ion spray source operated in the positive ionization mode. The source parameters were collision energy 40 eV, collision gas 6 psi, Nebulizer Gas 1, Turbo IonSpray Gas 2, and curtain gas 35 psi. Quantification was obtained by using multiple reaction monitoring mode of the transitions of mass to charge ratio [m/z] 510.3 →417 for DCC‐2618 (ripretinib), m/z 496.2 →403 for DP‐5439, m/z 515.3 →417 for DCC‐2618‐d_5_, and m/z 501.2 →403 for DP‐5439‐d_5_.

Following the bioanalytical method validation guidance, the analytical method was validated to verify that the assay was a robust and reliable measurement of ripretinib and DP‐5439 in human plasma with dipotassium ethylenediaminetetraacetic acid (K2EDTA) as the anticoagulant. The linear range of method was 2.00–1000 ng/mL for both ripretinib and DP‐5439. The coefficient of variation (CV%) (precision) for intra‐ and interassay at each quality control (QC) level was ≤15.0% (≤20.0% at the LLOQ QC level: 2 ng/mL for both ripretinib and DP‐5439). The mean % Bias (accuracy) at each QC level was within ±15.0% (±20.0% at the LLOQ QC level) from its respective nominal concentration. Ripretinib and DP‐5439 have been demonstrated to be stable in human plasma with K2EDTA as the anticoagulant for four cycles of freeze (−20 and −70°C) and thaw (room temperature) in the presence or absence of concomitant medications (itraconazole/hydroxyitraconazole, pantoprazole, or rifampin) and 16 hours at room temperature under normal laboratory lighting in the presence or absence of concomitant medications (itraconazole/hydroxyitraconazole, pantoprazole, or rifampin).

### Pharmacokinetic Assessments

Plasma concentration–time data were analyzed using noncompartmental methods using Phoenix^®^ WinNonlin^®^ v7.0 (Certara USA Inc., Princeton, New Jersey). Derived PK parameters included area under the concentration–time curve from time 0 to time t (AUC_0–t_) and area under the concentration–time curve from 0 to infinity (AUC_0–∞_), maximum observed concentration (C_max_), time of maximum observed concentration (t_max_), elimination half‐life (t_1/2_), apparent systemic clearance, and for DP‐5439, metabolite to parent ratio for AUC_0–t_, AUC_0–∞_, and C_max_.

The PK population included all participants who received ≥1 dose ripretinib and had ≥1 nonmissing PK concentration in plasma reported for ripretinib or DP‐5439. The PK evaluable set included all participants in the PK population who had ripretinib PK parameters on both ripretinib dosing days, bioanalytical results for ≥75% of planned analyses, no missed ripretinib doses and ≤2 missed perpetrator (itraconazole, pantoprazole, or rifampin) doses before the second dosing day of ripretinib, and no emesis within 8 hours of ripretinib administration.

### Safety Assessments

Safety was assessed with treatment‐emergent adverse events (TEAEs) coded using Medical Dictionary for Regulatory Activities (MedDRA) v21.1 and graded per National Cancer Institute Common Terminology Criteria for Adverse Events (CTCAE) v5.0, clinical laboratory results, vital signs, 12‐lead ECGs, and physical examinations. TEAEs were defined as adverse events (AEs) that occurred/worsened during or after administration of any of the study drugs. Relationship to study drug was assessed by the investigator. The safety population included all enrolled participants who received ≥1 dose of ripretinib.

### Statistical Methods

Participant demographics, clinical characteristics, and PK parameters were summarized using descriptive statistics. Safety data were summarized as n (%). For the evaluation of each DDI, an analysis of variance was performed using log‐transformed data for C_max_, AUC_0–t_, and AUC_0–∞_ for both ripretinib and DP‐5439, with treatment (ripretinib with or without itraconazole, pantoprazole, or rifampin) as a fixed effect. The geometric least‐squares (LS) mean ratios and corresponding 90% confidence intervals (CI) of C_max_, AUC_0–t_, and AUC_0–∞_ were calculated. PK parameters were calculated using Phoenix^®^ WinNonlin^®^ v7.0 or higher (Certara USA Inc.). No DDI was concluded if the 90%CI of the ratio of the test to the reference was completely within the range of 0.80–1.25 for C_max_, AUC_0–t_, and AUC_0–∞_.

## Results

### Participant Demographics

Participants were healthy volunteers (Table 1). In the itraconazole study, 20 participants were enrolled and one participant discontinued treatment with itraconazole on day 14 due to a TEAE of drug eruption on day 14 (ripretinib was received on day 11; the participant did not receive the last three doses of itraconazole). However, the participant received ripretinib on days 11 and 7 out of 10 scheduled itraconazole doses, including the dose on day 14, and had blood samples collected until 168 hours postdose. Therefore, the PK data for this participant were included in the PK analysis, and all 20 participants were included in the PK evaluable set for summary statistics. At enrollment, mean age was 32.6 years (range 19–55 years) and mean BMI was 24.3 kg/m^2^ (range 20.0–29.1 kg/m^2^); 45% of participants were female and 75% were white.

**Table 1:** Participant Baseline Demographics in the Safety Population

Demographic Characteristics	Ripretinib + Itraconazole (N = 20)	Ripretinib + Rifampin (N = 24)	Ripretinib + Pantoprazole (N = 25)
Age, years	32.6 ± 10.3	37.3 ± 11.3	33.6 ± 13.6
BMI, kg/m^2^	24.3 ± 2.8	25.8 ± 2.4	24.8 ± 3.2
Height, cm	171.6 ± 9.5	172.9 ± 11.0	174.5 ± 8.9
Weight, kg	71.8 ± 11.7	77.5 ± 13.2	76.0 ± 13.7
Sex, n (%)			
Female	9 (45.0)	10 (41.7)	13 (52.0)
Male	11 (55.0)	14 (58.3)	12 (48.0)
Race, n (%)			
American Indian or Alaska Native	1 (5.0)	1 (4.2)	0
Black or African American	2 (10.0)	4 (16.7)	1 (4.0)
White	15 (75.0)	17 (70.8)	23 (92.0)
Other	2 (10.0)^a^	2 (8.3)^b^	1 (4.0)^c^

BMI, body mass index; SD, standard deviation.

Data are mean ± SD, unless otherwise indicated.

^a^ One participant reported Caucasian/Native American, one participant reported Black and White.

^b^ Race not specified.

^c^ One participant reported Black and White.

In the rifampin study, 24 participants were enrolled and received a dose of ripretinib 100 mg on day 1. A total of three participants discontinued the study drug due to TEAEs before the second dose of ripretinib and therefore were excluded from PK evaluable analysis. Overall, 21 participants were included in the PK evaluable set for summary statistics. At enrollment, mean age was 37.3 years (range 22–54 years) and mean BMI was 25.8 kg/m^2^ (range 21.8–29.9 kg/m^2^). Participants were 41.7% female and 70.8% white.

In the pantoprazole study, 25 participants were enrolled and 23 completed all study treatments; 23 participants were included in the PK evaluable set for summary statistics. Two participants were excluded from the PK analysis because no PK blood samples were collected after 48 hours postdose. Both subjects discontinued during Period 1. One participant discontinued the study due to a TEAE of vomiting and one participant withdrew. The majority of participants were female (52.0%) and white (92.0%). At enrollment, mean age was 33.6 years (range 18–53 years) and mean BMI was 24.8 kg/m^2^ (range 18.5–29.9 kg/m^2^).

### Pharmacokinetics

#### Itraconazole (Strong CYP3A Inhibitor)

**Figure 2:**
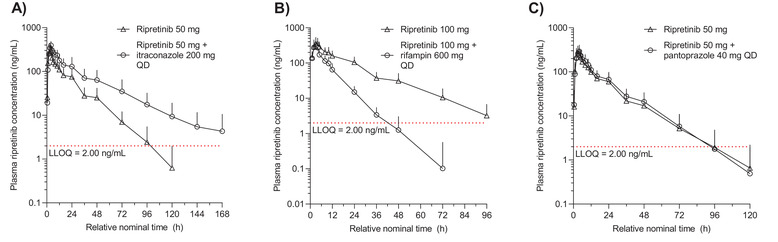
Mean plasma ripretinib concentration‐time profiles for drug interaction assessments for (A) itraconazole, (B) rifampin, and (C) pantoprazole on semi‐logarithmic scales. Data are mean ± SD. Data at 144 and 168 hours are not shown in (C) due to the small sample size. LLOQ, lower limit of quantitation; QD, once daily; SD, standard deviation.

In the itraconazole study, all 20 participants were included in the PK evaluable population. Exposure to ripretinib was increased when coadministered with itraconazole (Figure 2A). Mean concentration–time profiles of ripretinib declined in a multi‐exponential manner after reaching peak concentration and remained above the LLOQ until 96 hours postdose for ripretinib alone and up to 168 hours postdose for ripretinib with itraconazole. The median t_max_ for oral ripretinib 50 mg with and without itraconazole was 4.0 and 3.0 hours, respectively, and geometric mean t_1/2_ was 24 and 16 hours, respectively (Table 2). Higher geometric mean AUC_0–t_ (8060 vs 4064 ng·h/mL) and AUC_0–∞_ (8306 vs 4179 ng·h/mL) were observed for ripretinib 50 mg with vs without itraconazole. Geometric mean C_max_ was 395 ng/mL for ripretinib 50 mg with itraconazole, compared with 291 ng/mL for ripretinib 50 mg alone.

**Table 2:** Plasma PK Parameters of Ripretinib in the PK Evaluable Set

	Ripretinib + Itraconazole		Ripretinib + Rifampin		Ripretinib + Pantoprazole	
PK Parameters	Ripretinib 50 mg (N = 20)	Ripretinib 50 mg + Itraconazole 200 mg QD (N = 20)	Ripretinib 100 mg (N = 21)	Ripretinib 100 mg + Rifampin 600 mg QD (N = 21)	Ripretinib 50 mg (N = 23)	Ripretinib 50 mg + Pantoprazole 40 mg QD (N = 23)
AUC_0–t_ (ng·h/mL)						
Geometric mean (CV%)	4064 (43)	8060 (47)	5720 (39)	2231 (36)	3610 (39)	3935 (39)
Arithmetic mean (SD)	4405 (1802)	8897 (4288)	6617 (2326)	2364 (837)	3869 (1562)	4216 (1723)
AUC_0–∞_ (ng·h/mL)						
Geometric mean (CV%)	4179 (42)	8306 (48)	5825 (39)	2268 (35)	3690 (38)	4034 (38)
Arithmetic mean (SD)	4505 (1786)	9191 (4455)	6225 (2365)	2400 (839)	3953 (1589)	4312 (1739)
C_max_ (ng/mL)						
Geometric mean (CV%)	291 (50)	395 (23)	363 (39)	299 (40)	289 (31)	298 (38)
Arithmetic mean (SD)	321 (142)	404 (88)	392 (182)	321 (126)	301 (90)	319 (125)
t_max_ (h), median (min, max)	3 (2, 8)	4 (3, 5)	3 (2, 5)	3 (2, 4)	4 (2, 5)	4 (2, 8)
t_1/2_ (h)						
Geometric mean (CV%)	16 (33)	24 (47)	15 (23)	6 (46)	13 (40)	14 (26)
Arithmetic mean (SD)	17 (6)	27 (11)	15 (3)	7 (5)	14 (6)	14 (3)
CL/F (L/h)						
Geometric mean (CV%)	12 (42)	6 (48)	17 (39)	44 (35)	14 (38)	12 (38)
Arithmetic mean (SD)	13 (5)	7 (3)	18 (7)	47 (16)	14 (5)	13 (5)

AUC_0–∞_, area under the concentration–time curve from time 0 and extrapolated to infinity; AUC_0–t_, area under the concentration–time curve from time 0 to time t; CL/F, apparent systemic clearance; C_max_, maximum observed concentration; CV%, coefficient of variation; PK, pharmacokinetic; QD, once daily; SD, standard deviation; t_1/2_, elimination half‐life; t_max_, time of maximum observed concentration.

**Figure 3:**
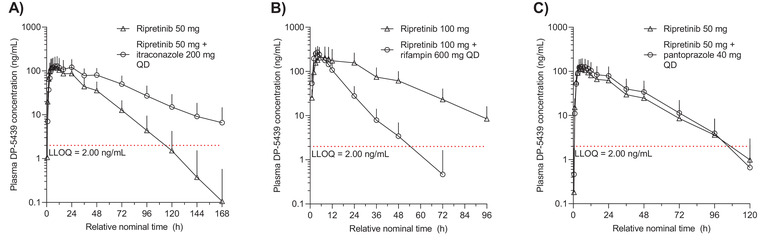
Mean plasma DP‐5439 concentration–time profiles for drug interaction assessments for (A) itraconazole, (B) rifampin, and (C) pantoprazole on semi‐logarithmic scales. Data are mean ± SD. Data at 144 and 168 hours are not shown in (C) due to the small sample size. LLOQ, lower limit of quantitation; QD, once daily; SD, standard deviation.

DP‐5439, the active metabolite of ripretinib, had similar mean plasma concentration–time profiles as ripretinib with and without itraconazole (Figure 3A). After reaching peak plasma concentrations, DP‐5439 remained above the LLOQ for up to 96 hours postdose for ripretinib alone and up to 168 hours for ripretinib with itraconazole. Median t_max_ for plasma DP‐5439 for ripretinib 50 mg with and without itraconazole was 9 and 5 hours, respectively, and geometric mean terminal t_1/2_ was 29 and 17 hours, respectively (Table 3). For DP‐5439, higher geometric mean AUC_0–t_ (6884 vs 3541 ng·h/mL) and AUC_0–∞_ (7277 vs 3651 ng·h/mL) were observed for ripretinib 50 mg with vs without itraconazole.

**Table 3:** Plasma PK Parameters of DP‐5439 in the PK Evaluable Set

	Ripretinib + Itraconazole		Ripretinib + Rifampin		Ripretinib + Pantoprazole	
PK Parameters	Ripretinib 50 mg (N = 20)	Ripretinib 50 mg + Itraconazole 200 mg QD (N = 20)	Ripretinib 100 mg (N = 21)	Ripretinib 100 mg + Rifampin 600 mg QD (N = 21)	Ripretinib 50 mg (N = 23)	Ripretinib 50 mg + Pantoprazole 40 mg QD (N = 23)
AUC_0–t_ (ng·h/mL)						
Geometric mean (CV%)	3541 (78)	6884 (75)	6294 (58)	2727 (55)	2539 (98)	3325 (75)
Arithmetic mean (SD)	4263 (2435)	8000 (3490)	7325 (4828)	3046 (1332)	3241 (2051)	4012 (2406)
AUC_0–∞_ (ng·h/mL)						
Geometric mean (CV%)	3651 (76)	7277 (73)	6504 (59)	2767 (54)	2622 (95)	3412 (73)
Arithmetic mean (SD)	4367 (2471)	8432 (3752)	7565 (4911)	3086 (1342)	3313 (2062)	4089 (2403)
C_max_ (ng/mL)						
Geometric mean (CV%)	114 (65)	121 (66)	187 (52)	256 (44)	109 (55)	122 (50)
Arithmetic mean (SD)	133 (80)	142 (85)	215 (160)	276 (104)	122 (53)	136 (68)
t_max_ (h), median (min, max)	5 (3, 24)	9 (6, 48)	5 (4, 24)	4 (2, 5)	4 (4, 24)	6 (4, 24)
t_1/2_ (h)						
Geometric mean (CV%)	17 (22)	29 (40)	16 (18)	7 (35)	15 (37)	15 (29)
Arithmetic mean (SD)	18 (4)	31 (12)	17 (3)	7 (3)	15 (5)	15 (4)
CL/F (L/h)						
Geometric mean (CV%)	13 (76)	7 (73)	15 (59)	35 (54)	19 (95)	14 (73)
Arithmetic mean (SD)	17 (18)	9 (11)	17 (9)	40 (23)	28 (39)	18 (15)
M:P AUC_0–t_						
Geometric mean (CV%)	0.9 (69)	0.9 (81)	1.1 (55)	1.3 (38)	0.7 (79)	0.9 (61)
Arithmetic mean (SD)	1.1 (0.7)	1.1 (0.8)	1.3 (0.7)	1.3 (0.5)	0.9 (0.5)	1.0 (0.5)
M:P AUC_0–∞_						
Geometric mean (CV%)	0.9 (67)	0.9 (77)	1.1 (55)	1.3 (38)	0.7 (76)	0.9 (60)
Arithmetic mean (SD)	1.1 (0.7)	1.1 (0.7)	1.3 (0.7)	1.3 (0.5)	0.9 (0.4)	1.0 (0.5)
M:P C_max_						
Geometric mean (CV%)	0.4 (51)	0.3 (63)	0.5 (45)	0.9 (25)	0.4 (53)	0.4 (40)
Arithmetic mean (SD)	0.5 (0.3)	0.4 (0.3)	0.6 (0.2)	0.9 (0.2)	0.4 (0.2)	0.5 (0.2)

AUC_0–∞_, area under the concentration–time curve from time 0 and extrapolated to infinity; AUC_0–t_, area under the concentration–time curve from time 0 to time t; CL/F, apparent systemic clearance; C_max_, maximum observed concentration; M:P, metabolite to parent ratio; QD, once daily; PK, pharmacokinetic; SD, standard deviation; t_1/2_, elimination half‐life; t_max_, time of maximum observed concentration.

**Figure 4:**
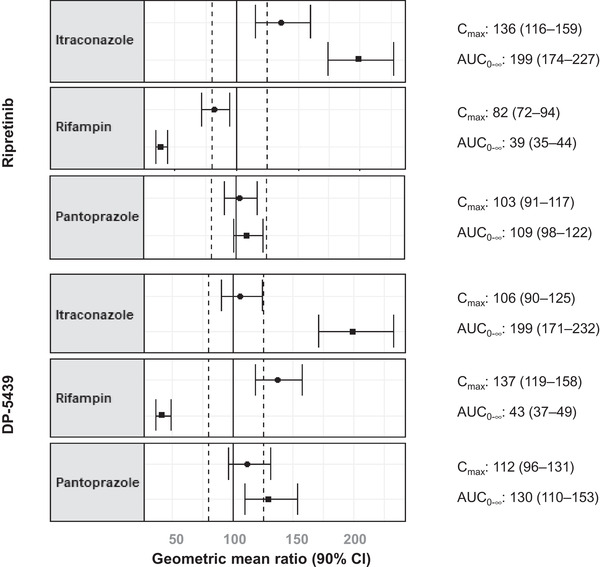
Forest plot summarizing the effect of coadministered drug in ripretinib and DP‐5439 pharmacokinetics with corresponding geometric mean ratios for C_max_ and AUC_0–∞_ (90%CI). Ratios are for ripretinib + drug coadministration compared to ripretinib dosing alone. Dotted lines represent the 80%–125% range. AUC_0‐∞_, area under the concentration–time curve from time 0 and extrapolated to infinity; CI, confidence interval; C_max_, maximum observed concentration.

Statistical comparisons supported increased exposure of ripretinib and DP‐5439 when ripretinib was coadministered with itraconazole. When comparing ripretinib with itraconazole to ripretinib alone, geometric LS mean ratios for ripretinib AUC_0–t_ and AUC_0–∞_ were 198% and 199%, respectively, and the C_max_ ratio was 136% (Figure 4). Geometric LS mean ratios for DP‐5439 were 194% and 199% for AUC_0–t_ and AUC_0–∞_, respectively, whereas the C_max_ ratio was 106%.

#### Rifampin (Strong CYP3A Inducer)

In the rifampin study, 21 of 24 participants were included in the PK evaluable set, as three participants discontinued treatment prior to day 18. Exposure to ripretinib 100 mg was decreased when coadministered with rifampin, a strong CYP3A4 inducer. Both with and without rifampin, the mean concentration–time profiles of ripretinib declined in a multi‐exponential manner after reaching the peak concentration; it remained above the LLOQ for up to 96 hours postdose for ripretinib alone and for up to approximately 36 hours postdose for ripretinib with rifampin (Figure 2B). Median t_max_ of ripretinib was 3 hours both with and without rifampin, and geometric mean t_1/2_ was lower for ripretinib with rifampin compared to ripretinib alone (7 vs 15 hours) (Table 2). Lower geometric mean AUC_0–t_ was also observed for ripretinib with rifampin compared to ripretinib alone (2231 vs 5720 ng·h/mL), as was AUC_0–∞_ (2268 vs 5825 ng·h/mL). Geometric mean C_max_ for plasma ripretinib was also lower for ripretinib with rifampin (299 ng/mL) compared to ripretinib alone (363 ng/mL).

Similarly, exposure to DP‐5439 was reduced for ripretinib 100 mg with rifampin compared to ripretinib 100 mg alone. The mean concentration–time profile of DP‐5439 declined in a multi‐exponential manner after reaching peak plasma concentration and remained above the LLOQ up to 96 hours postdose for ripretinib alone and up to approximately 48 hours postdose for ripretinib with rifampin (Figure 3B). Median t_max_ of DP‐5439 was 5 hours for ripretinib alone and 4 hours for ripretinib with rifampin; geometric mean t_1/2_ was 16 and 7 hours, respectively (Table 3). Ripretinib with rifampin resulted in lower plasma DP‐5439 geometric mean AUC_0–t_ (2727 ng·h/mL) and AUC_0–∞_ (2767 ng·h/mL) compared with ripretinib alone (6294 and 6504 ng·h/mL, respectively). However, geometric mean C_max_ of DP‐5439 was 256 ng/mL for ripretinib with rifampin and 187 ng/mL for ripretinib alone.

Ratios of geometric LS means for plasma ripretinib AUC_0–t_, AUC_0–∞_, and C_max_ were 39%, 39%, and 82%, respectively, for ripretinib with rifampin relative to ripretinib alone (Figure 4). Ratios of geometric LS means for plasma DP‐5439 AUC_0–t_, AUC_0–∞_, and C_max_ were 43%, 43%, and 137%, respectively, for ripretinib with rifampin relative to ripretinib alone.

#### Pantoprazole (Proton Pump Inhibitor)

Of the 25 participants in the pantoprazole study, 23 were included in the PK evaluable set; two participants were excluded from the PK analysis because no blood samples were collected after 48 hours postdose. There was no difference in the mean plasma ripretinib concentration–time profile between ripretinib 50 mg alone and ripretinib administered with the PPI pantoprazole (Figure 2C). Both with and without pantoprazole, plasma ripretinib declined in a multi‐exponential manner after peak concentration and remained above the LLOQ until just prior to 96 hours postdose. Median t_max_ was 4 hours for ripretinib 50 mg both with and without pantoprazole, and geometric mean t_1/2_ was 14 and 13 hours, respectively (Table 2). Administration of ripretinib with pantoprazole resulted in a slightly higher geometric mean AUC_0–t_ of 3935 ng·h/mL compared with 3610 ng·h/mL for ripretinib alone, and geometric mean AUC_0–∞_ was 4034 and 3690 ng·h/mL for ripretinib with and without pantoprazole, respectively. Geometric mean C_max_ was comparable for ripretinib with and without pantoprazole (298 and 289 ng/mL, respectively).

For both ripretinib with and without pantoprazole, mean concentration–time profiles of DP‐5439 declined in a mono‐exponential manner after peak concentration, remaining above the LLOQ just prior to 96 hours postdose (Figure 3C). Median t_max_ for plasma DP‐5439 for ripretinib with and without pantoprazole was 6 and 4 hours, respectively, and geometric mean terminal t_1/2_ was 15 hours for both (Table 3). Higher plasma DP‐5439 was observed with ripretinib with pantoprazole compared with ripretinib alone for geometric mean AUC_0–t_ (3325 vs 2539 ng·h/mL) and AUC_0–∞_ (3412 vs 2622 ng·h/mL). Geometric mean DP‐5439 C_max_ was also slightly higher for ripretinib with pantoprazole (122 ng/mL) compared with ripretinib alone (109 ng/mL).

For ripretinib with pantoprazole relative to ripretinib alone, the ratios of geometric LS mean for AUC_0–t_, AUC_0–∞_, and C_max_ were 109%, 109%, and 103%, respectively, within the 80%–125% range (Figure 4). Ratios of geometric LS means for plasma DP‐5439 AUC_0–t_, AUC_0–∞_, and C_max_ were 131%, 130%, and 112%, respectively, for ripretinib with pantoprazole relative to ripretinib alone.

### Safety

Overall, in the itraconazole study, 14 (70%) participants experienced a total of 44 TEAEs (Table 4). Per the investigator, 13 (65%) of these experienced 36 TEAEs attributed to study drug. No TEAEs led to study discontinuation; one (5.0%) participant discontinued itraconazole treatment on day 14 due to a TEAE of drug eruption, assessed by the investigator as possibly related to ripretinib and/or itraconazole. No deaths occurred and no participants experienced serious AEs (SAEs).

**Table 4:** TEAEs Reported by ≥2 Participants in any Treatment Group (Safety Population)

SOC Preferred Term	Ripretinib + Itraconazole (N = 20)	Ripretinib + Rifampin (N = 24)	Ripretinib + Pantoprazole (N = 25)
Participants reporting any TEAE	14 (70.0)	24 (100)	22 (88.0)
Diarrhea	4 (20.0)	12 (50.0)	6 (24.0)
Abdominal pain	3 (15.0)	0	4 (16.0)
Abdominal pain lower	1 (5.0)	1 (4.2)	2 (8.0)
Constipation	1 (5.0)	1 (4.2)	2 (8.0)
Nausea	2 (10.0)	7 (29.2)	5 (20.0)
Vomiting	1 (5.0)	4 (16.7)	1 (4.0)
Abdominal discomfort	0	3 (12.5)	0
Lipase increase	5 (25.0)	11 (45.8)	5 (20.0)
Amylase increase	4 (20.0)	10 (41.7)	4 (16.0)
ALT increase	1 (5.0)	1 (4.2)	2 (8.0)
AST increase	0	0	2 (8.0)
Headache	2 (10.0)	14 (58.3)	8 (32.0)
Fatigue	1 (5.0)	2 (8.3)	0

AE, adverse event; ALT, alanine aminotransferase; AST, aspartate aminotransferase; SOC, system organ class; TEAE, treatment‐emergent AE.

Data are n (%). TEAEs were defined as AEs that occurred/worsened during/after administration of any of the study drugs. AEs were coded using MedDRA v21.1. Participants experiencing multiple AEs in a category were counted only once for that category.

In the rifampin DDI study, all 24 (100%) participants experienced 94 TEAEs (Table 4); 23 (95.8%) experienced 78 TEAEs assessed as related to study drug per the investigator. No deaths or SAEs occurred and no participants discontinued the study.

Overall, in the pantoprazole study, 22 (88%) participants experienced a total of 63 TEAEs (Table 4). Of these, 19 (76%) experienced 42 TEAEs attributed to study drug per the investigator. A Grade 2 event of vomiting on day 1 resulted in treatment discontinuation and subsequent study discontinuation. No deaths occurred and no participants experienced SAEs.

## Discussion

Given that patients receiving ripretinib as treatment for GIST are continuously dosed, understanding the potential of concomitant medications to affect exposure to ripretinib is particularly important to ensure the efficacy and safety of their treatment. Incubations with recombinant human CYPs and human liver microsome indicated that the metabolism of ripretinib is primarily mediated by CYP3A4/5, forming 1 active metabolite, DP‐5439 (also metabolized via CYP3A4/5, per in vitro observations) (data on file). Based on the in vitro studies, coadministration of ripretinib with a strong CYP3A inhibitor or inducer may have a potential for an increase or decrease in systemic exposure to ripretinib and DP‐5439 because of the inhibition or induction of CYP3A‐mediated intestinal and/or hepatic metabolism of ripretinib. In the studies described herein, we evaluated the effect of coadministration of the strong CYP3A inhibitor itraconazole and the strong CYP3A inducer rifampin on the PK of ripretinib and its active metabolite DP‐5439 in healthy adult participants. The effect of ARA (pantoprazole, a PPI) on the PK of ripretinib and DP‐5439 was also evaluated due to ripretinib's pH‐dependent solubility.

Because ripretinib is mainly metabolized by CYP3A, it was anticipated that ripretinib exposure would increase significantly with coadministration of ripretinib with itraconazole, a well‐characterized strong inhibitor of CYP3A. In the current study, itraconazole was administered as an oral solution on an empty stomach to ensure the highest oral absorption under fasting conditions and minimize gastrointestinal tract irritation during fasting. The 3‐day lead‐in ensured that CYP3A was maximally inhibited prior to coadministration with ripretinib. Daily itraconazole coadministration resulted in increased exposure to ripretinib and its active metabolite, DP‐5439, by up to 99%. These results suggest that itraconazole inhibits the metabolism of ripretinib and possibly DP‐5439 as well, confirming the anticipated interaction. The tolerability of higher exposure is supported by previous safety and PK data.^7^, ^10^ In a phase 1 study of ripretinib in patients with cancer, the maximum tolerated dose was not reached with doses up to ripretinib 200 mg twice daily.^7^ Dose escalation to ripretinib 150 mg twice daily was offered to patients in a phase 1 and phase 3 clinical study after radiologic disease progression on 150 mg once daily.^7^, ^11^ This regimen had a similar safety profile as seen at 150 mg once daily and provided benefit in progression‐free survival.^10^, ^12^ Steady‐state PK exposure following ripretinib 150 mg twice daily was approximately 2‐fold higher compared with ripretinib 150 mg once daily.^7^, ^10^ Generally, ripretinib demonstrated flat exposure–safety relationships in clinical studies conducted in patients with cancer. No exposure–response relationships were observed with parent, metabolite DP‐5439, or combined analytes except for any grade myalgia and palmar‐plantar erythrodysesthesia, for which ripretinib trough concentrations were higher in participants with these AEs. However, the exposure–safety relationships were relatively shallow, and most AEs were Grades 1 and 2. In addition, concentration‐corrected QT interval analysis did not suggest particular concern across a range of plasma concentration of either ripretinib or DP‐5439 (data on file). Overall, existing safety data indicate that the 2‐fold higher exposures observed with the prototype strong inhibitor of CYP3A/P‐gp may not raise a particular safety concern; however, more frequent monitoring is recommended for adverse reactions, as individual patient exposure may vary with different strong inhibitors of CYP3A, such as ritonavir or cobicistat, which may be more potent CYP3A inhibitors compared to itraconazole.^13^, ^14^

We also evaluated ripretinib 100 mg in combination with rifampin 600 mg once daily, a strong CYP3A inducer. Exposure to ripretinib was reduced when coadministered with rifampin, as anticipated. When combined with rifampin, ripretinib and DP‐5439 exposure was less than half of that of ripretinib alone. These data indicate that rifampin induces metabolism of both ripretinib and DP‐5439. The decrease in overall exposure to both ripretinib and DP‐5439 is likely due to an increase in the biotransformation rate, as shown by the increase in the rate of appearance of DP‐5439. Our data suggest that strong CYP3A inducers should not be used concomitantly with ripretinib, as decreased ripretinib exposure may result in reduced antitumor activity. Prescribers should refer to approved labeling for specific dose recommendation for concomitant use of strong and moderate CYP3A inducers.

As ripretinib has pH‐dependent solubility (ie, reduced solubility at neutral pH), we also assessed ripretinib in combination with the PPI pantoprazole as a representative gastric ARA. PPIs are expected to represent the worst‐case scenario regarding potential interference with intestinal absorption of weakly basic drugs with pH‐dependent solubility.^9^ We found that pantoprazole 40 mg once daily had no effect on exposure to ripretinib, but exposure to DP‐5439 was increased by 12%–30%. These data indicate ripretinib absorption is insensitive to potent ARA, such that patients can coadminister ripretinib with ARAs without dose adjustment. While the mechanism for the increased exposure to DP‐5439 with coadministration of pantoprazole is unknown, the magnitude of increased exposure to the metabolite DP‐5439 only is unlikely to be clinically relevant given the safety data described above.

In each DDI evaluation, the safety of ripretinib was manageable and no new safety signals were identified. The ripretinib doses evaluated in each study were lower than those used clinically to reduce the potential for AEs in healthy volunteers.

## Conclusions

The strong CYP3A inhibitor itraconazole significantly increased systemic exposure of ripretinib and its active metabolite DP‐5439, whereas the strong CYP3A inducer rifampin significantly reduced the exposure of ripretinib and its active metabolite DP‐5439. Hence, patients should be monitored more frequently for adverse reactions when a strong CYP3A inhibitor is coadministered with ripretinib. Decreased exposure of ripretinib may decrease antitumor activity, therefore concomitant use of ripretinib with strong CYP3A inducers should be avoided. Prescribers should refer to approved labeling for specific dose recommendations with concomitant use of strong and moderate CYP3A inducers. The gastric acid reducer pantoprazole 40 mg once daily had no effect on exposure to ripretinib, but exposure to DP‐5439 was increased by 12%–30%. The magnitude of increased exposure to the metabolite DP‐5439 only is unlikely to be clinically relevant. Single‐dose ripretinib, administered with or without itraconazole, rifampin, or pantoprazole, appeared to be generally safe and well tolerated in these healthy adult volunteers.

## Data Sharing Statement

Qualified scientific and medical researchers can make requests for individual participant data that underlie the results reported in this article, after de‐identification, at info@deciphera.com. Proposals for data will be evaluated and approved by Deciphera at its sole discretion. All approved researchers must sign a data access agreement before accessing the data. Data will be available as soon as possible but no later than within 1 year of the acceptance of the article for publication and for 3 years after article publication. Deciphera will not share data from identified participants or a data dictionary.

## Conflicts of Interest

X.L. and R.R.‐S. are employees and stockholders of Deciphera Pharmaceuticals, LLC. J.M. and J.W. were employees of Deciphera Pharmaceuticals, LLC at the time of study conduct and may hold stock. M.J.S. is an employee of Certara, a consulting firm in integrated drug development, and has directly consulted with a variety of not‐for‐profit global health organizations, biotechnology companies, and pharmaceutical companies.
